# A comparative study on predicting influenza outbreaks using different feature spaces: application of influenza-like illness data from Early Warning Alert and Response System in Syria

**DOI:** 10.1186/s13104-020-4889-5

**Published:** 2020-01-16

**Authors:** Ali Darwish, Yasser Rahhal, Assef Jafar

**Affiliations:** Department of Informatics, Higher Institute for Applied Sciences and Technology, Damascus, Syria

**Keywords:** Influenza-like illness (ILI), Feature space, Time series analysis, Long short term memory (LSTM)

## Abstract

**Objective:**

An accurate forecasting of outbreaks of influenza-like illness (ILI) could support public health officials to suggest public health actions earlier. We investigated the performance of three different feature spaces in different models to forecast the weekly ILI rate in Syria using EWARS data from World Health Organization (WHO). Time series feature space was first used and we applied the seven models which are Naïve, Average, Seasonal naïve, drift, dynamic harmonic regression (Dhr), seasonal and trend decomposition using loess (STL) and TBATS. The Second feature space is like some state-of-the-art, which we named $53-weeks-before\_52-first-order-difference$ feature space. The third one, we proposed and named $n-years-before\_m-weeks-around$ (YnWm) feature space. Machine learning (ML) and deep learning (DL) model were applied to the second and third feature spaces (generalized linear model (GLM), support vector regression (SVR), gradient boosting (GB), random forest (RF) and long short term memory (LSTM)).

**Results:**

It was indicated that the LSTM model of four layers with $1-year-before\_4-weeks-around$ feature space gave more accurate results than other models and reached the lowest MAPE of $3.52\%$ and the lowest RMSE of 0.01662. I hope that this modelling methodology can be applied in other countries and therefore help prevent and control influenza worldwide.

## Introduction

Influenza epidemic results in three to five million cases of severe illnesses and approximately 290,000 to 650,000 deaths worldwide each year [1]. WHO’s Early Warning, Alert and Response System (EWARS) is designed to improve disease outbreak detection in emergency settings. The system has been built in SYRIA since 2012 to collect and detect near real-time information on several outbreaks including influenza [2]. The Existing researches on modeling influenza epidemic falls into two categories: Mechanistic and Statistical models. They are summarized in literature reviews [3–6] and in the CDC comparisons [7, 8]. Researches under statistical category vary according to the different Features and methods used. Some researchers used the number of patients in the past as features [9–13], while others integrated other data sources to predict the number of patients in the future. Examples of these sources are climatological data [14, 15], search engine queries [16–19], public comments on social media like Twitter [20, 21], online information-seeking behavior on websites like Wikipedia [22, 23] and a combination of multiple data streams [15, 24–26]. Different methods on these features were applied. Some researches treated the problem as an instance of more general time series forecasting using time series methods (ARIMA, ARIMA-STL, GARMA) [9, 10, 17, 27], while others used ML methods including Stacked linear regression [24, 26], AdaBoost regression with decision trees [26], GB [12], SVR [26, 28], elastic net [28] RF [11, 12, 28], Artificial Neural Network (ANN) [12, 20]. Recently, a DL method Called LSTM has attracted much interest in ILI prediction and gave excellent results, which are more accurate than those of other methods [12, 13, 15, 29]. In addition to investigating the performance of the three different feature spaces with multiple time-series, ML and DL based methods to predict the weekly ILI rate in Syria; we proposed novel future spaces $n-years-before\_m-weeks-around$ that integrate into state-of-the-art ML and DL methods. There are two important contributions of this paper. First, the use of $n-years-before\_m-weeks-around$ future spaces to predict ILI rate. Second, analyzing multiple models performance over the EWARS data from WHO in Syria.

## Main text

### Materials and methods

#### Data

We collected SYRIA flu data from the EWARS reports published by WHO on the website [30]. We only used the Flu Data from the first week of 2014 to the 42nd week of 2018. To avoid any possible population variations, we adopted the ILI rates as predictors (x) and responses (y) of our models.

$${\text{ILI\, rate}} = {\text{ILI\,number}} / {\text{Total\,number\,of \, illnesses}}$$

**Fig. 1:**
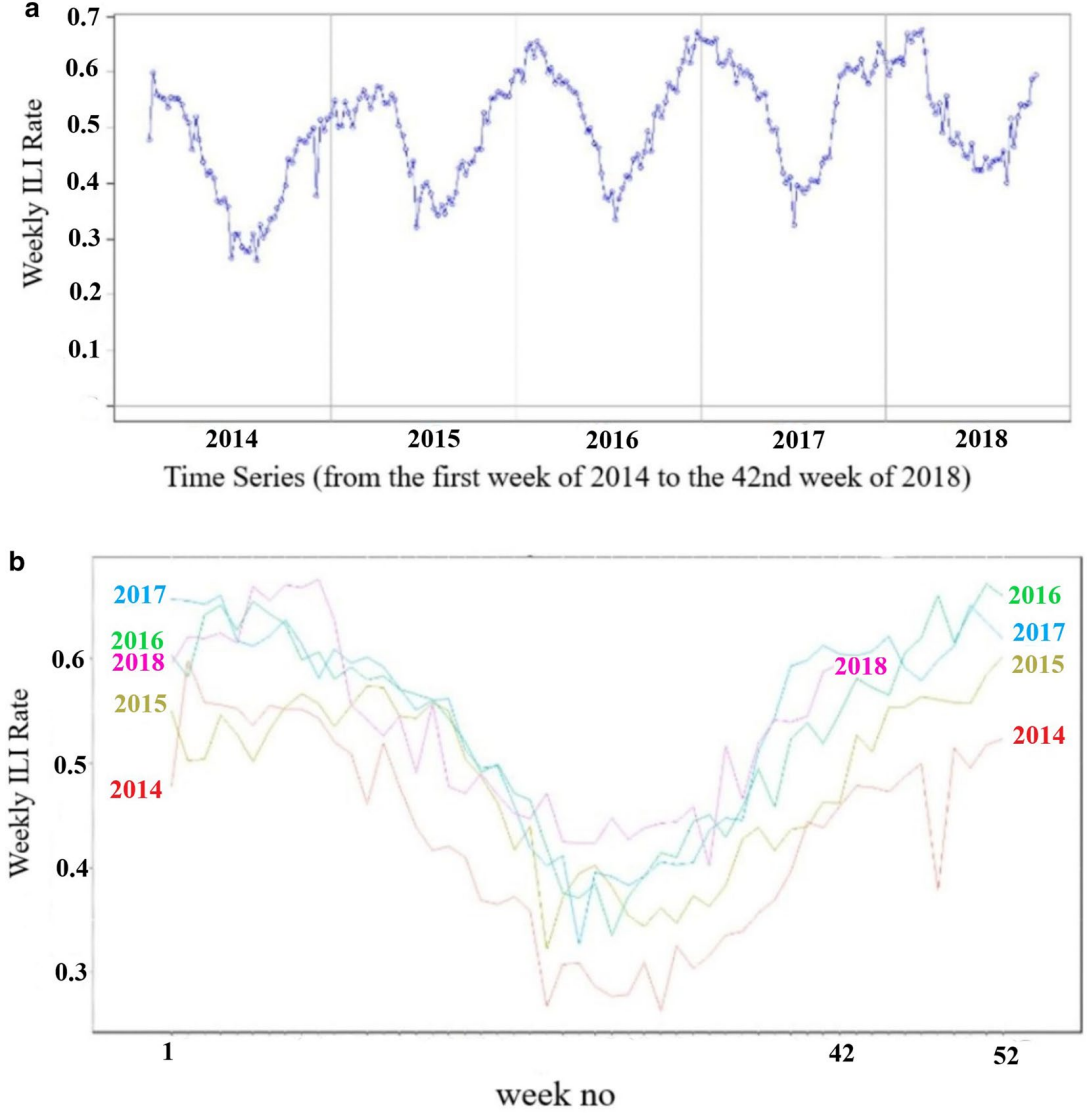
**a** The ILI rate in SYRIA from the first week of 2014 to the 42nd week of 2018; The Y-axis represents the weekly ILI rate, and the X-axis represents the time series. **b** The ILI rate in SYRIA from the first week of 2014 to the 42nd week of 2018 (seasonal plot); The Y-axis represents the weekly ILI rate, and the X-axis represents the weeks

**Fig. 2:**
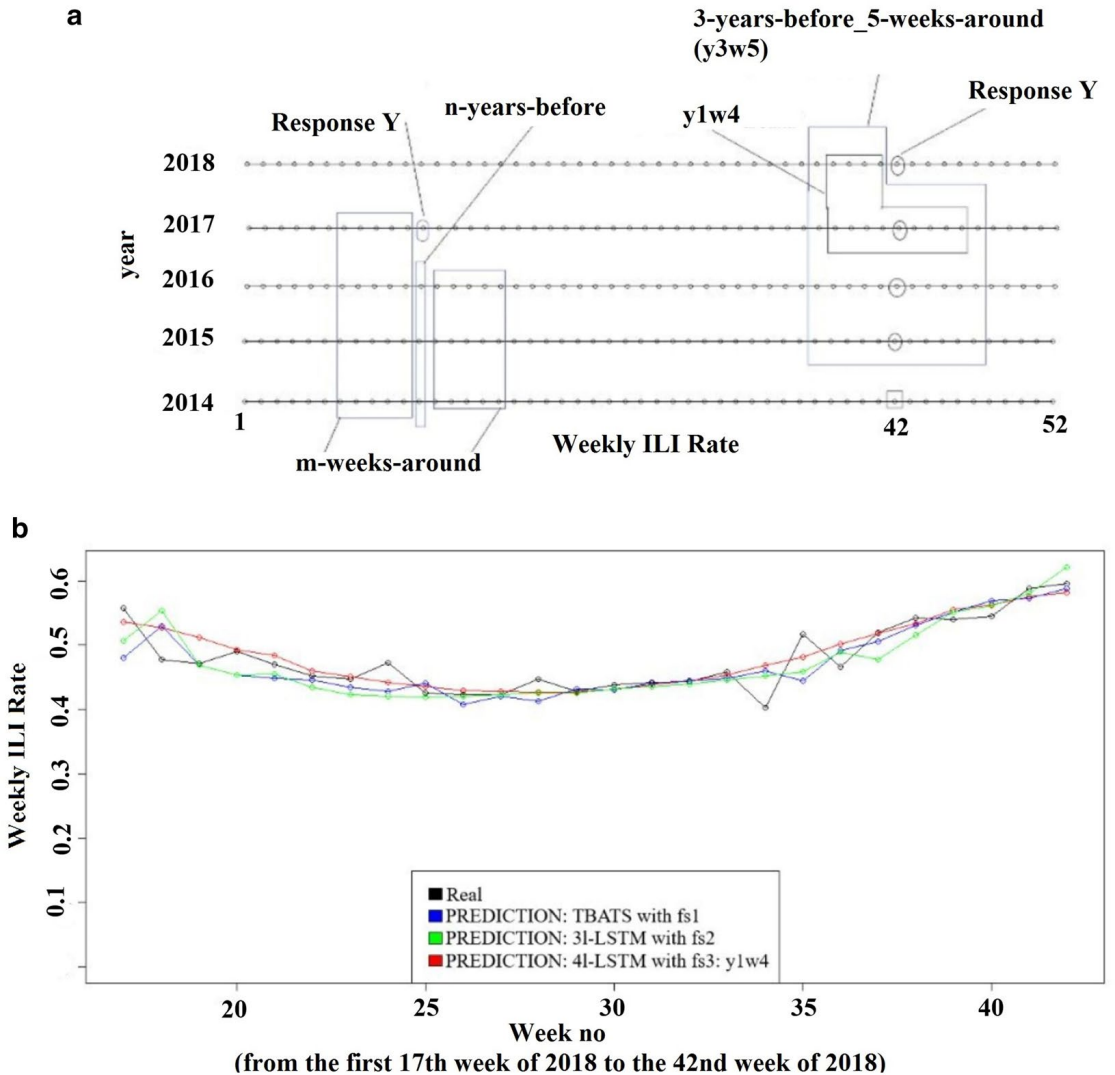
**a** $n-years-before\_m-weeks-around (YnWm)$ feature spaces. **b** The real and prediction of weekly ILI rate of the testing set for TBATS with fs1: time series feature space, LSTM of 3 layers with fs2: $53- weeks-before\_52-first-order-differences$ feature space, LSTM of 4 layers with fs3: $n-years-before\_m-weeks-around (y1w4)$ feature space

Figure 1a illustrates the raw data. The Y-axis represents the weekly ILI rate, and the X-axis represents the time series. The seasonality is obvious, as shown in Fig 1b. We split the data into two parts: the first 90% was the training set and the last 10% was the testing set.

#### Feature space

In this study, we reviewed three types of feature space. We named them as (time series feature space, $53-weeks-before\_52-first-order-difference$ feature space and $n-years-before\_m-weeks-around$ feature space). Tables S1 and S2 illustrate the data set, response, predictors and the pretreatment of the source data. An additional pdf file shows this in more detail (see Additional file 1).

*Time series feature space (fs1)* We treated the ILI weekly data as time series with seasonal value 52.

$53- weeks-before\_52-first-order-differences$*feature space (fs2)* Some previous studies found that using the ILI rate of the past 53 weeks and the 52 first-order differences helped improve the results of the prediction models for influenza data [11–13].

$n-years-before\_m-weeks-around$*(YnWm) feature spaces (fs3)* We reviewed a maximum of 3 years before and 5 weeks around. To predict the ILI rate of week WX of year YX, We used the ILI rate of the past m weeks before WX in YX, the ILI rate of past n seasonal weeks and the ILI rate of the m weeks around seasonal weeks. In the case of $1-year-before\_4-weeks-around$, the response y is the ILI rate of week 30 of year 2017 then the predictors are the ILI rates of weeks 29, 28, 27, 26 from year 2017 and the ILI rates of weeks 26, 27, 28, 29, 30, 31, 32, 33, 34 from year 2016. The number of predictors and rows changed depending on the value of n and m and we had to drop some data rows so that we get the same training data length for any value of n and m. Figure 2a illustrates the feature spaces with examples of $3-years-before\_5-weeks-around$ and $1-year-before\_4-weeks-around$ feature spaces.

#### Models

The models, programming languages, and libraries, which were used in this study were illustrated in Additional file 1. We trained all models in R Programming Language (version 3.4.4). For time series models, we used the “forecast” package (version 8.4). For ML models, we applied the caret package (Version 6.0-8). For DL models, we used the Keras package (Version 2.2.4) based on Tensorflow (Version 1.10). A personal computer with Intel I7-8550U processor, 8 GB of RAM and an NVIDIA 130 MX GPU was used for the experiments. Each experiment takes approximately 1 to 30 min to train the model. The prediction takes less than 5 s on the same hardware.

#### Metrics

We compared different models with different feature spaces using the mean absolute percentage error (MAPE) and root mean squared error (RMSE) as key performance indicators (KPIs).

$$\begin{aligned} {\text{MAPE}}& = \frac{1}{n} \sum _{t=1}^n 100\, \left| \frac{F_t-A_t}{A_t}\right| \% \\ {\text{RMSE}} &= \sqrt{\frac{1}{n} \sum _{t=1}^n (F_t-A_t)^2} \end{aligned}$$

where At is the actual value and Ft is the forecasted value.

### Results

#### fs1 with time series models

Seven methods (Average, Naïve, Seasonal Naïve, Drift, STL, DHR and TBATS) were applied in a recursive way. We trained the model on the training data set then predicted the ILI rate of week number x then we repeated the process by combining the actual ILI rate of week number x with the training data set. The metrics of all models are presented in Table  1(a), (b). it was indicated that the TBATS model outperformed other six models in predicting weekly ILI rate (MAPE = 4.66%, RMSE = 0.03096). Our explanation of the success of TBATS model is that the seasonality is allowed to change slowly over time in a TBATS model, while DHR, STL, Seasonal Naïve models force the seasonal patterns to repeat periodically without changing and Average, Naïve, Drift models do not depend on the seasonal patterns. We considered TBATS model a baseline in comparing the results of the following models.

**Table 1:** The MAPEs and RMSEs of the testing set for all methods applied on fs1: time series and fs2: $53- weeks-before\_52-first-order-differences$ and fs3: $n-years-before\_m-weeks-around$ feature space

(a) The MAPEs%							
Models	Average	Naïve	Seasonal naïve	Drift	STL	DHR	TBATS
Features:fs1	11.31	5.69	7.9	5.7	5.72	5.12	*4.66*
Models	GLM	SVR	GB	RF	3-Layers-LSTM	4-Layers-LSTM	
Features:fs2	6.16	5.83	5.94	5.89	*4.72*	4.9	
Models	GLM	SVR	GB	RF	3-Layers-LSTM	4-Layers-LSTM	
Features:fs3							
Y0W1	5.69	5.68	6.75	6.86	5.66	5.67	
Y0W2	5.76	5.72	7.1	6.3	5.34	5.38	
Y0W3	5.73	5.73	6.69	6.29	5.36	5.27	
Y0W4	5.49	5.61	6.92	6.19	5.44	5.3	
Y0W5	5.68	5.71	7.29	5.91	5.47	5.39	
Y1W0	6.48	6.72	6.89	9.27	4.5	4.12	
Y2W0	6.17	5.85	6.47	8.91	4.67	4.6	
Y3W0	5.96	6.4	8.37	9.08	4.82	4.69	
Y1W1	*5.12*	5.23	6.36	6.94	3.94	4.8	
Y1W2	5.61	*5*	5.26	6.48	4.09	4.09	
Y1W3	6.22	5.27	6.22	6.89	3.97	3.63	
Y1W4	6.22	5.3	5.7	7.11	*3.89*	*3.52*	
Y1W5	6.08	5.23	6.98	6.18	4.46	3.54	
Y2W1	5.26	5.32	5.56	7.22	4.44	4.03	
Y2W2	6.01	5.08	*4.24*	*5.78*	4.22	4.3	
Y2W3	7.43	5.68	6.19	6.17	4.43	3.99	
Y2W4	7.33	5.4	6.71	6.36	4.81	4.39	
Y2W5	7.23	5.34	5.53	6.44	4.61	4.71	
Y3W1	6.01	5.43	6.65	6.9	4.72	4.37	
Y3W2	6.86	5.49	5.75	6.22	5.5	4.74	
Y3W3	7.74	5.76	6.18	6.34	6.21	5.42	
Y3W4	8.24	5.92	6.97	6.24	6.43	5.92	
Y3W5	8.56	6.14	6.24	6.48	6.78	6.13	

(b) The RMSEs							
Models	Average	Naïve	Seasonal naïve	Drift	STL	DHR	TBATS
Features:fs1	0.05796	0.03925	0.04454	0.03931	0.03672	0.03179	*0.03096*
Models	GLM	SVR	GB	RF	3-Layers-LSTM	4-Layers-LSTM	
Features:fs2	0.03743	0.03643	0.03687	0.03699	*0.02294*	0.0237	
Models	GLM	SVR	GB	RF	3-Layers-LSTM	4-Layers-LSTM	
Features:fs3							
Y0W1	0.03852	0.03866	0.04389	0.04245	0.02744	0.02767	
Y0W2	0.03933	0.03923	0.04525	0.03868	0.02598	0.02593	
Y0W3	0.03754	0.03861	0.04286	0.03915	0.02631	0.02549	
Y0W4	0.03689	0.03755	0.04355	0.0392	0.02666	0.02566	
Y0W5	0.03686	0.03798	0.04528	0.03825	0.02611	0.02565	
Y1W0	0.03781	0.03874	0.04163	0.04927	0.02113	0.01985	
Y2W0	0.03685	0.03534	0.04096	0.04761	0.02252	0.02189	
Y3W0	0.03629	0.03965	0.04526	0.05006	0.02312	0.02323	
Y1W1	*0.03341*	0.03361	0.03851	0.0396	0.01929	0.02296	
Y1W2	0.03654	*0.03272*	0.02968	0.03826	0.01938	0.0198	
Y1W3	0.03742	0.03309	0.03434	0.03768	0.01873	0.01763	
Y1W4	0.03745	0.03265	0.03369	0.03851	*0.01825*	*0.01662*	
Y1W5	0.03652	0.03175	0.04083	0.0335	0.02139	0.01682	
Y2W1	0.03472	0.034	0.03515	0.04029	0.02108	0.01984	
Y2W2	0.03886	0.03352	*0.0248*	*0.03327*	0.01994	0.02276	
Y2W3	0.04222	0.03381	0.03401	0.03705	0.02094	0.01926	
Y2W4	0.04159	0.03249	0.03787	0.03909	0.02288	0.02122	
Y2W5	0.04276	0.03291	0.03502	0.03929	0.02148	0.02276	
Y3W1	0.0362	0.03305	0.03476	0.03988	0.02253	0.02121	
Y3W2	0.04038	0.03156	0.03382	0.03729	0.02659	0.02319	
Y3W3	0.04366	0.03357	0.03517	0.03822	0.0313	0.02588	
Y3W4	0.04539	0.03405	0.04228	0.03822	0.03226	0.02919	
Y3W5	0.05098	0.03592	0.03747	0.03928	0.03388	0.03064	

Italics number indicate best result

#### fs2 with ML and DL models

We applied four ML methods (GLM, SVR, GB and RF) and two DL methods (LSTM with 3 layers and LSTM with 4 layers). Table 1(a), (b) shows that none of the models achieved better results than the baseline model in MAPE metric but in RMSE metric, the LSTM model of 3 layers (RMSE = 0.02294) outperformed the baseline model and all otehr models with fs1 and fs2 .

#### fs3 with ML AND DL models

According to $0<=n<=3\ and\ 0<=m<=5$ we trained 23 feature spaces in four ML methods (GLM, SVR, GB and RF) and two DL methods (LSTM with 3 layers and LSTM with 4 layers). Totally, we made 138 experiments with results shown in Table  1(a), (b). In accordance with MAPE metric, We found that 22 experiments show a decrease in metrics less than that of the baseline model (1 experiment: by using GB model with Y2W2 feature space, 10 experiments: by using LSTM model of 3 layers with “Y1Wm, Y2Wm: excluding m=0 and m=4” feature spaces, 11 experiments: by using LSTM model of 4 layers with “Y1Wm: excluding m=1,Y2Wm: excluding m=5,Y3w1” feature spaces) but In accordance with RMSE metric, We found that 44 experiments show a decrease in metrics less than that of the baseline model (1 experiment: by using GB model with Y2W2 feature space, 20 experiments: by using LSTM model of 3 layers with “YnWm: excluding n=3 with m=3,4,5” feature spaces, 23 experiments: by using LSTM model of 4 layers with “YnWm” feature spaces). we achieved the best result (MAPE = 3.52%, RMSE = 0.01662) by using LSTM model of 4 layers with Y1W4 feature space. in comparison to models with fs2, the results show that for any model used, there are at least one value to n and m in fs3 that achieved better result than the same model with fs3. Our explanation of the success of this feature space is that models can learn the seasonality by n years before and can recognize the trend of the data by m weeks around.

The temporal variation of the real weekly ILI rate and the predicted values obtained from the three models (the best result in each feature space) for the test period were plotted in Fig 2b. As can be seen, the predicted values of weekly ILI rate were in a good agreement with their related observed values and the used models could be used to model the weekly ILI rate. Moreover, LSTM model of 4 layers with fs3 resulted in better predicted values and trend between them for the observed values of ILI rate than the other models especially when there is a change in trend between observations.

### Discussion

The accurate forecast of an outbreak of ILI could support public health officials in taking public health actions, such as allocating or temporarily readjusting medical resources for hospitals and medical centers. The ILI rate varies from year to year depending on multiple factors. Despite this difference, the ILI rate plot in each year takes a semi-constant form with an increase or decrease compared to previous years and could be predicted by using specific observations of past years with statistical models. Performance of statistical models is data dependent and there is no model that performs well in all situations. Therefore, evaluating the performance of different models is of great importance as they provide useful and important information regarding strengths and weaknesses of the models and gives an insight to use better models for forecasting purposes. Some sate of the arts [11, 12] utilize 53 weeks before with 52 first order difference with different statistical models and found that both the recent observations and the later observations with the difference were interesting and had significant influence on the predication. In this study, we proposed novel future spaces, namely $n-years-before\_m-weeks-around$, and compared to some existing future spaces that utilize historical observations in different ways by integrating it into state-of-the-art ML and DL models. Our results revealed the success of our future space for some values of n and m and its failure for other values to outperform other future spaces in prediction ILI rate in Syria. This fact suggests that combining carefully selected number of Nearby Historical Observations with Carefully selected number of seasonal Historical Observations is advantageous over simply choosing all Historical Observations. While the results presented here are for ILI within Syria, our novel future spaces shows promise to be easily extended to accurately track not only influenza in other countries but also other infectious diseases Through careful tuning of the values of n and m. We believe that it is up to policymakers in organizations concerned with EWARS to decide whether these forecasts are ready for use in decision support at the current level of accuracy.

In conclusion, we performed Naïve, Average, Seasonal naïve, Drift, DHR, STL, TBATS, GLM, SVR, GB, RF and LSTM methods with different feature spaces to predict the weekly ILI rate using EWARS data from WHO in Syria. We found that the TBATS method with time series feature space gave better results than those resulted from all methods in $53-weeks-before\_52-first-order-differences$ feature space. We also found that the GLM, SVR, GB, RF and LSTM methods with a good choice to n and m in $n-years-before\_m-weeks-around$ feature space gave better results than those resulted in $53-weeks-before\_52-first-order-differences$ feature space. In all the models, the LSTM model of 4 layers reached the lowest MAPE (3.52%) and the lowest RMSE (0.01662).

## Limitations

Climatological data and Pharmaceutical Sales could be used to achieve better performance of the used models. We would like to investigate the impact of these parameters in future work.

## Supplementary information

**Additional file 1.** Details about feature spaces and programing languages and libraries. .pdf file with three tables: the first table explaint the $53- weeks-before\_52-first-order-difference$ feature space, the second table explaint the $N-years-before\_m-weeks-around$ feature space and the third one show the models, programming languages, and libraries, which were used in this study.

**Additional file 2.** Dataset used in manuscript. .csv file contains the EWARS data about ILI in SYRIA from the first week of 2014 to the the 42nd week of 2018.

## Data Availability

The data is provided as Additional file 2. The data is also publically available on: http://www.emro.who.int/syr/publications-other/ewars-weekly-bulletin-2014.html, http://www.emro.who.int/syr/publications-other/ewars-weekly-bulletin-2015.html, http://www.emro.who.int/syr/information-resources/2016-ewars-bulletins.html, http://www.emro.who.int/syr/ewars-workshops/ewars-bulletins-2017.html, http://www.emro.who.int/syr/information-resources/ewars-weekly-bulletins-2018.html.

## Abbreviations

EWARS: Early Warning Alert and Response System
Dhr: dynamic harmonic regression
STL: seasonal and trend decomposition using Loess
YnWm: $n-years-before\_m-weeks-around$ feature space
ML: machine learning
DL: deep learning
GLM: generalized linear model
SVR: support vector regression
GB: gradient boosting
RF: random forest
LSTM: long short term memory
MAPE: mean absolute percentage error
RMSE: root mean square error
