# Supplementary material for: A comparative study on predicting influenza outbreaks using different feature spaces: application of influenza-like illness data from Early Warning Alert and Response System in Syria
Source: BMC Res Notes. 2020 Jan 16;13:33. doi: 10.1186/s13104-020-4889-5 (PMC6964210; doi:10.1186/s13104-020-4889-5)
Supplement: Supplementary file 1 — Additional file 1. Details about feature spaces and programing languages and libraries. .pdf file with three tables: the first table explaint the $53- weeks-before\_52-first-order-difference$53-weeks-before_52-first-order-difference feature space, the second table explaint the $N-years-before\_m-weeks-around$N-years-before_m-weeks-around feature space and the third one show the models, programming languages, and libraries, which were used in this study. [file 13104_2020_4889_MOESM1_ESM.pdf]

53- weeks-before _52-first-order-difference feature space. In all, we have 105 predictors.

Feature space									
Time series		53- weeks-before _52-first-order-differences							
		Response	Current data & historic data				First-order difference		
Year	Weeks (W)	ILI rates "y"	ILI rates "x1" (1 W ago)	ILI rates "x2" (2 W ago)	…	ILI rates "x53" (53 W ago)	(1 W ago) " x54" (2 W ago)	…	(1 W ago) " x105" (53 W ago)
2014	1	0.47833	N/A	N/A	…	N/A	N/A	…	N/A
…	…	…	…	…	…	…	…	…	…
2015	2	0.5028	0.54994	0.52333	…	0.47833	0.02661	…	0.07161
…	…	…	…	…	…	…	…	…	…
2018	41	0.58767	0.54521	0.53932	…	0.59863	0.00589	…	-0.05342
2018	42	0.59545	0.58767	0.54521	…	0.61305	0.04246	…	-0.02538

N-years-before_m-weeks-around feature space. In case of n=1 and m=5, we have 38 predictors for use as feature spaces.

Feature space										
Time series		n-years-before_m-weeks-around								
		Response	Current data & historic data ILI rates							
Year	Weeks (W)	ILI rates "y"	"x1" 1 W Before "y"	…	"x5" 5 W Before "y"	"x6" 5 W before "x11"	…	"x11" 52 W before "y"	…	"x16" 5 W After "x11"
2014	1	0.47833	N/A	… …	N/A	N/A	…	N/A	…	N/A
…	…	…	…	…	…	…	…	…	…	…
2017	6	0.61254	0.61788	…	0.6575	0.60343	…	0.65494	…	0.58054
…	…	…	…	…	…		…	…	…	…
2018	41	0.58767	0.54521	…	0.4658	0.44534	…	0.61305	…	0.59173
2018	42	0.59545	0.58767	…	0.5195	0.51195	…	0.60443	…	0.57901

The models, programming languages, and libraries, which were used in this study

Feature space	*models*	*Programming Libraries*
Time series	Average, Naïve, Seasonal Naïve, Drift, STL, DHR, TBATS	Forecast (version 8.4)
53- weeks-before_52-first-order-differences	GLM, SVR, GB, RF	Caret package (Version 6.0-8)
	LSTM	Keras (Version 2.2.4) Tensorflow (Version 1.10)
n-years-before_m-weeks-around	GLM, SVR, GB, RF	Caret package (Version 6.0-8)
	LSTM	Keras (Version 2.2.4) Tensorflow (Version 1.10)
